# Case Report: Dural infiltration of intervertebral disc material mimicking spinal cord meningioma in a dog

**DOI:** 10.3389/fvets.2025.1621529

**Published:** 2025-07-01

**Authors:** Karin Sakamoto, Yukiko Nakano, James K. Chambers, Yui Kobatake, Masao Mizuno, Hiroaki Kamishina

**Affiliations:** ^1^Faculty of Applied Biological Sciences, Joint Department of Veterinary Medicine, Gifu University, Gifu, Japan; ^2^The Animal Medical Center of Gifu University, Gifu University, Gifu, Japan; ^3^Laboratory of Veterinary Pathology, Graduate School of Agricultural and Life Sciences, The University of Tokyo, Tokyo, Japan; ^4^The United Graduate School of Veterinary Sciences, Gifu University, Gifu, Japan; ^5^Mizuno Pet Clinic, Gifu, Japan; ^6^KyotoAR Advanced Veterinary Medical Center, Kyoto, Japan

**Keywords:** dog, intervertebral disc herniation, dural infiltration, MRI, hemilaminectomy

## Abstract

A six-year-old mixed-breed dog presented with a two-month history of peracute back pain and right hindlimb monoparesis. Neurological examination revealed reduced proprioceptive positioning of the right hindlimb, and palpation of the L2–L4 area elicited a pain response. Magnetic resonance imaging revealed a contrast-enhanced mass extending to the dura mater, located on the right ventral side of the spinal cord at the level of the L2–L3 vertebral bodies. A hemilaminectomy was performed to remove the mass. Histopathological examination revealed that the removed dura mater comprised intervertebral disc material that had infiltrated the dura mater. Postoperatively, the dog experienced temporary right hindlimb lameness, which resolved within 24 h. At three years and five months postoperatively, the dog was ambulatory without any neurological deficits or pain. This is the first report of dural infiltration of intervertebral disc material mimicking a spinal cord meningioma in a dog.

## 1 Introduction

Intervertebral disc (IVD) herniation is a common spinal disorder in dogs and typically manifests as either extrusion or protrusion of disk material (1). IVD extrusion (IVDE) often causes spinal cord compression and injury, resulting in neurological deficits, including acute neck or back pain, paresis, or plegia (1). Magnetic resonance imaging (MRI) is the most common imaging modality to diagnose IVDE (2, 3). MR images of patients with IVDE are characterized by extradural compression of the spinal cord over or near the IVD space (2). Intradural/intramedullary IVDE (IIVDE), in which disc materials are present in the subarachnoid space or spinal cord parenchyma, has also been reported in dogs; however, the infiltration of IVD material into the dura mater has not been reported (4–16). Here, we report our experience with a dog with dural infiltration of IVD material that mimicked a spinal cord meningioma on initial diagnostic imaging.

## 2 Case description

A 6-year-old intact male mixed-breed dog (Toy Poodle × Pomeranian), weighing 3.8 kg, was presented to a primary veterinarian with a history of peracute back pain. The dog was initially treated with firocoxib (3.5 mg/kg; Previcox57, Nippon Zenyaku Kogyo Co., Ltd., Fukushima, Japan) and omeprazole sodium (2.6 mg/kg; Omeprazole tablets 10 mg “TOWA,” Towa Pharma International Holdings, S. L., Osaka, Japan). Clinical signs improved over the next three days. However, a month later, the dog returned with recurrent back pain and right hindlimb monoparesis. Partial clinical improvement was observed with laser therapy (5 W, once or twice a week; DVL-20, ASUKA MEDICAL Inc., Kyoto, Japan) and medication treatment, including hypodermic injection of fuzapladib sodium hydrate (0.4 mg/kg; Brenda^®^ Z, Nippon Zenyaku Kogyo Co., Ltd., Fukushima, Japan), prednisolone (0.5–1.0 mg/kg; Prednisolone Inj. NZ, Nippon Zenyaku Kogyo Co., Ltd., Fukushima, Japan), lansoprazole (0.3 mg/kg; Takepron Intravenous 30 mg, Teva Takeda Yakuhin Ltd. Nagoya, Japan), and benzylpenicillin potassium (30,000 U/kg; Procaine Penicillin G in Aqueous Suspension for Veterinary Use, Riken Vets Pharma Inc., Saitama, Japan). Despite treatment, the back pain persisted.

Two months after the initial clinical onset, the dog was referred to the Animal Medical Center of Gifu University for further evaluation of neurological deficits. Upon physical examination, palpation of the L2–L4 area elicited a pain response. Further, neurological examination revealed reduced proprioceptive positioning of the right hindlimb with a normal gait. Spinal reflexes were within normal limits, and both mental status and cranial nerve examination findings were unremarkable. Hematological and plasma biochemical analyses revealed elevated blood urea nitrogen (48.7 mg/dL; range, 9.2–29.2 mg/dL) and total bile acid (38.9 μmol/L; range, 0.0–7.9 μmol/L). Thoracic and abdominal radiographs revealed microhepatica. Based on clinical findings, neuroanatomical diagnoses included the T3–L3 and L4–S3 spinal cord segments.

MRI of the thoracolumbar spine was performed with the dog in dorsal recumbency under general anesthesia using a high-field scanner (3.0 T; Achieva dStream, Philips, Amsterdam, Netherland). Anesthesia was induced with intravenous propofol (10 mg/kg; PropoFlo 28, Zoetis Japan, Tokyo, Japan) and maintained with isoflurane (2.0%; IsoFlo Zoetis Japan, Tokyo, Japan) in oxygen. The MRI sequences were acquired with 1.5 mm slice thickness, comprising T2 weighted images (T2WI, TR = 6,800, TE = 90 ms) and pre- and post-contrast enhanced T1 weighted images (T1WI and T1WI + C, TR = 600, TE = 20 ms) in sagittal, transverse, and dorsal planes. Gadodiamide hydrate (Omniscan, GE Healthcare Pharma, Tokyo, Japan) was administered intravenously at a dose of 0.1 mm/kg as the contrast medium.

**Figure 1:**
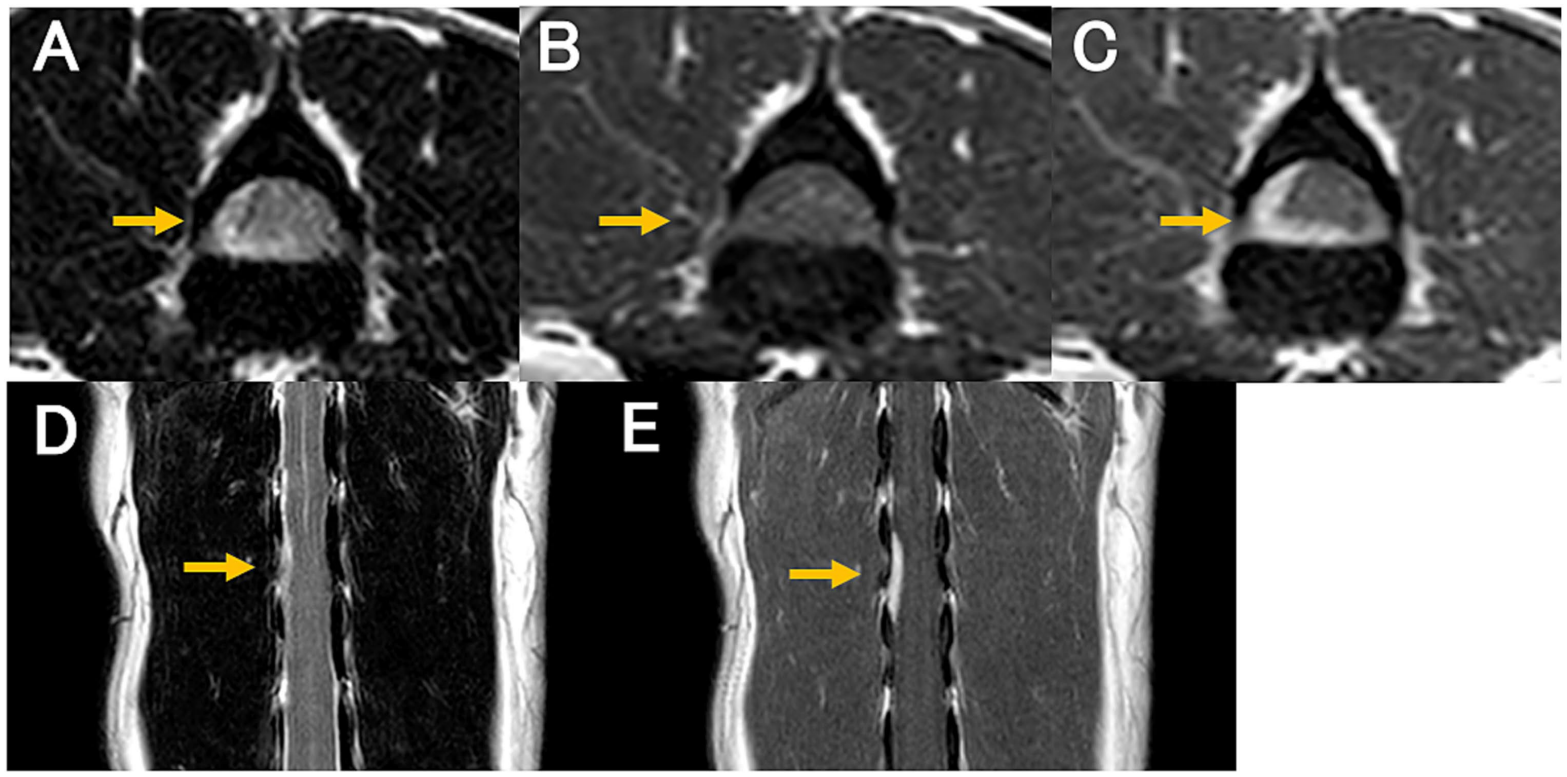
Magnetic resonance images at diagnosis. T2-weighted transverse **(A)**; pre-contrast T1-weighted transverse **(B)**; post-contrast T1-weighted transverse **(C)**; T2-weighted dorsal **(D)**; and post-contrast T1-weighted dorsal **(E)** images. A contrast-enhanced mass is continuous with the dura mater located on the right ventral side of the spinal cord at the level of L2 and L3 vertebral bodies (**C,E**; arrow). The mass appears isointense to hyperintense on the T2-weighted image **(A,D)** and isointense on the T1-weighted image **(B)**. The spinal cord compression by the mass is evident.

MRI revealed an isointense to hyperintense mass on T2WI, located on the right ventral side of the spinal cord at the level of the L2 and L3 vertebral bodies (Figures 1A,D). The mass appeared either extradural or intradural-extramedullary and caused mild spinal cord compression. The mass exhibited contrast-enhancement, appeared continuous with the dura mater, and was associated with partial dural thickening (Figures 1B,C,E). Computed tomography (CT; Alexion Advance, Canon Medical Systems, Tochigi, Japan) was performed for surgical planning and revealed no additional abnormalities.

Based on the signalment and clinical history, traumatic disease was considered the most likely differential diagnosis. Other differential diagnoses included neoplastic and inflammatory diseases. Considering the MRI findings of an isointense to hyperintense mass contiguous with the dura mater and clear contrast enhancement, meningioma was considered the first differential diagnosis.

**Figure 2:**
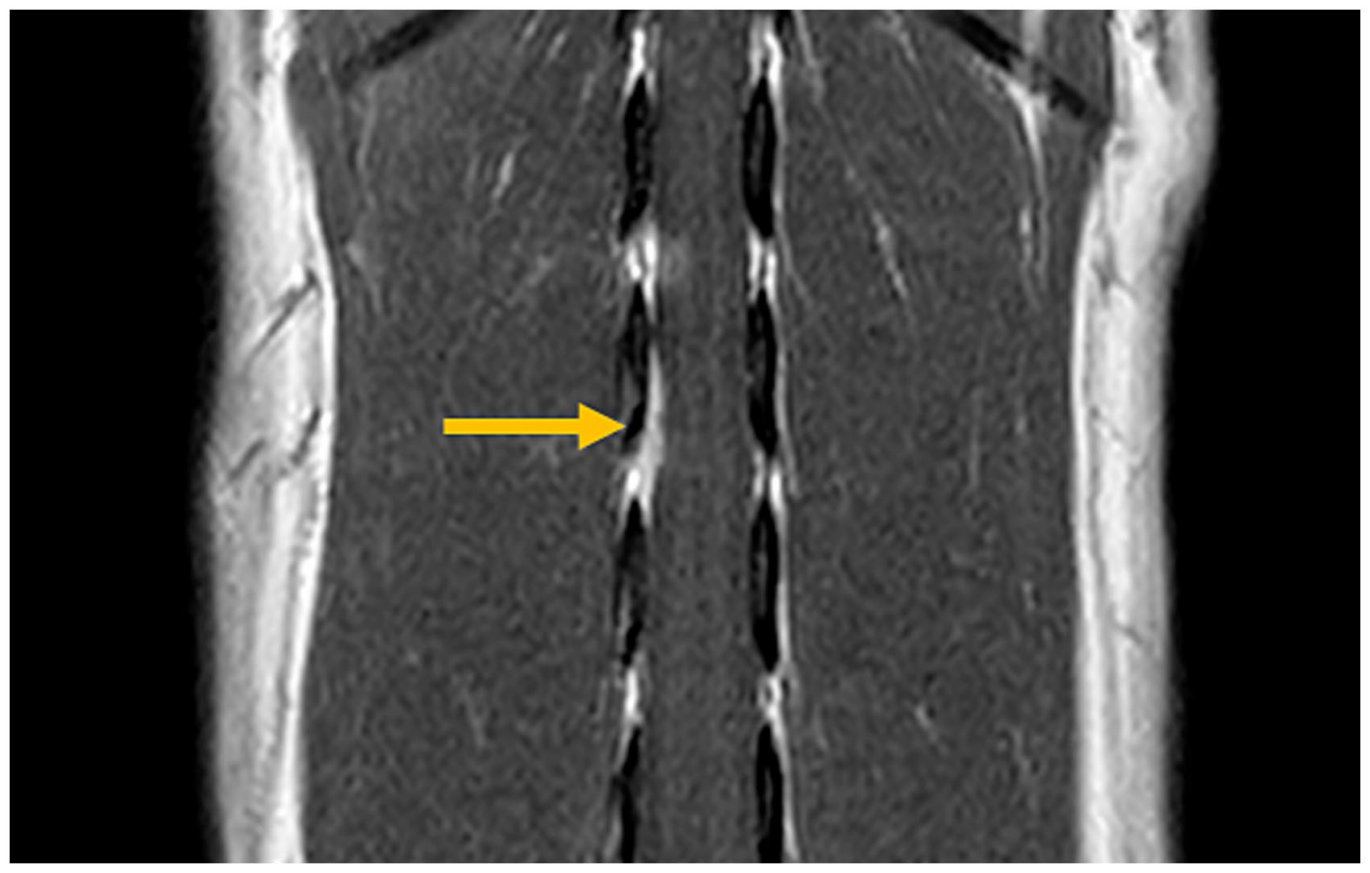
Magnetic resonance imaging prior to surgery (post-contrast T1-weighted transverse view). The previously identified contrast-enhanced mass is slightly reduced in size (arrow).

Surgical removal of the mass for spinal cord decompression and histopathological examination was performed two weeks after the initial presentation. Anesthesia was induced with propofol (8 mg/kg intravenously) and maintained with isoflurane (1.3–1.8%; IsoFlo, Zoetis Japan, Tokyo, Japan) in oxygen. The perioperative analgesia included continuous rate infusions of fentanyl citrate (2 μg/kg/h; Fentanyl Injection, Janssen Pharmaceutical K. K., Tokyo, Japan), remifentanil hydrochloride (15 μg/kg/h; Ultiva®, Janssen Pharmaceutical K. K., Tokyo, Japan), and ketamine hydrochloride (0.08 mg/kg/h; Ketalar®, Daiichi Sankyo Propharma Co., Ltd., Tokyo, Japan). The perioperative antibiotic therapy consisted of cefazolin (20 mg/kg intravenously; Cefamezin *α*, LTL Pharma, Tokyo, Japan) administered every 3 h during surgery. Blood pressure was managed with ephedrine (100 μg/kg intravenously; Ephedrin “NAGAI,” Nichi-iko Pharmaceutical, Toyama, Japan) in case of hypotension and atropine sulfate hydrate (10 μg/kg intravenously; Atropine Injection, TERUMO CORPORATION, Tokyo, Japan) for bradycardia. To induce muscle relaxation, continuous rate infusions of rocuronium bromide (0.5 mg/kg/h; ESLAX Intravenous, MSD, Tokyo, Japan) was administered. A second MRI was performed immediately before the surgery, revealing that the contrast-enhanced mass had slightly decreased in size (Figure 2). After the MRI, the dog was positioned in ventral recumbency for surgery. Right-sided hemilaminectomy was performed over the L2–L3 intervertebral space. Upon exposure, the spinal cord was visible, and the dura mater appeared thickened. For intraoperative lesion identification, fluorescein sodium (20 mg/kg intravenously; FLUORESCITE Intravenous Injection, Novartis Pharma K. K., Tokyo, Japan) was administered as previously described (17). The affected part of the dura mater was firmer than normal and exhibited a yellow discoloration after fluorescein sodium administration. The thickened dura mater was resected with surgical margins. The right L2 nerve root, which was stained yellow with fluorescein sodium, was also resected. An extradural, soft tissue material resembling nucleus pulposus was found beneath the spinal cord and excised. This material was not continuous with the dura mater. The thickened dura mater and right L2 nerve root were submitted for histopathological examination. During wound closure, sugammadex sodium (2 mg/kg intravenously; BRIDION Intravenous, MSD, Tokyo, Japan) was administered to reverse muscle relaxation. No intraoperative complications occurred, and the dog recovered uneventfully from general anesthesia. Postoperative management included continuous rate infusions of fentanyl citrate (1–3 μg/kg/h for 2 days) and ketamine hydrochloride (0.08 mg/kg/h for 2 h) for analgesia. Cefazolin (20 mg/kg, twice a day intravenously, 2 days) was administered as postoperative antibiotic therapy, along with prednisolone (0.5 mg/kg, subcutaneously, 1 day; Prednisolone Injection KS, Kyoritsu Seiyaku Corporation, Tokyo, Japan).

Immediately after the surgery, the dog experienced temporary right hindlimb lameness, which resolved by the following day. Three weeks postoperatively, the dog showed no neurological deficits, besides mildly reduced proprioceptive positioning in the right hindlimb. According to the reports from the primary veterinarian, the dog walked normally without any neurological deficits or pain at the time of its annual vaccination and 3 years and 5 months after surgery.

**Figure 3:**
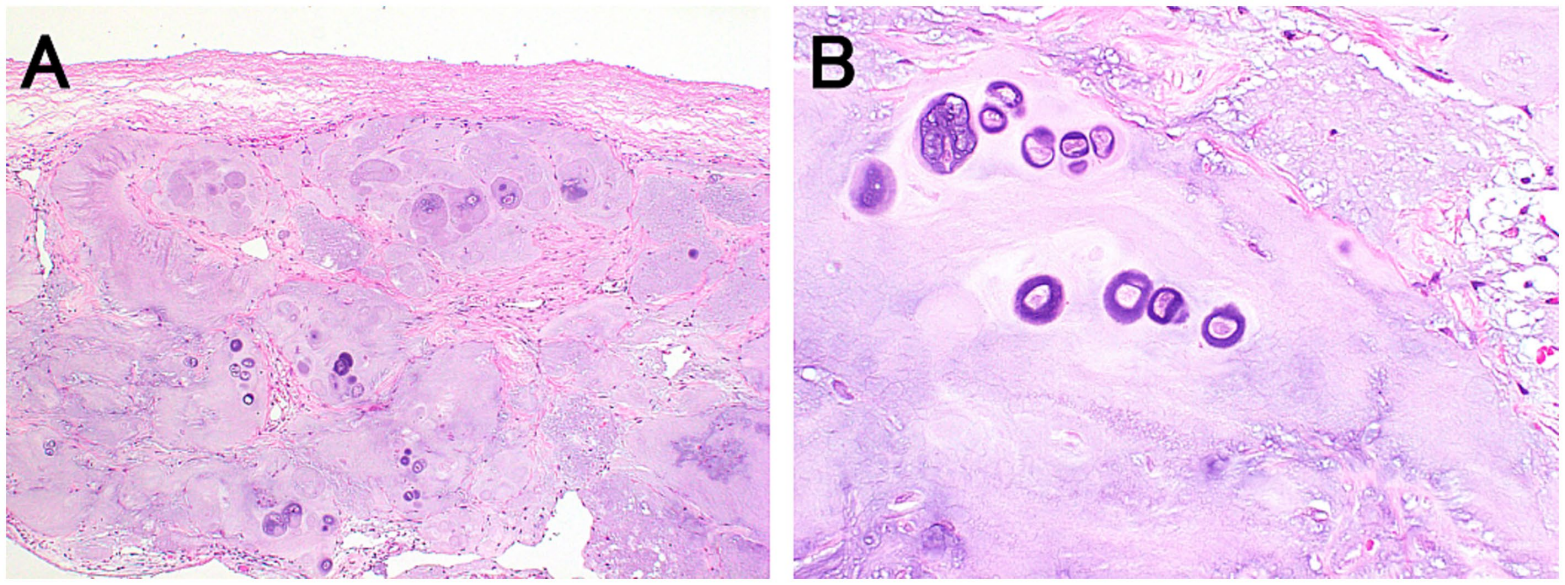
Histopathological findings of the thickened dura mater. Hematoxylin and eosin staining at low (**A**: ×40) and high (**B**: ×400) magnifications. The dura matter is thickened due to cartilage tissue in continuity with the dura mater **(A)**. The fibrous connective tissue surrounding the cartilage tissues is also observed. High magnification shows hyperplastic chondrocytes **(B)**.

Histopathological examination of the thickened dura mater revealed cartilage tissue in direct continuity with the dura mater (Figure 3). Surrounding the cartilage, connective tissue exhibited macrophage infiltration and hemosiderin pigmentation, suggesting intradural hemorrhage and inflammation. The axons of the right L2 nerve root were degenerated. No atypical cells suggestive of tumors were observed.

## 3 Discussion

IVD herniation is classically categorized as Hansen Types I or II herniation (18–20). Besides these traditional classifications, new subtypes have been reported based on the degree of spinal cord compression, localization, and properties of the disc material, including acute non-compressible nucleus pulposus extrusion (21), IIVDE (22), and acute compressive hydrated nucleus pulposus extrusion (23). In the present study, we report an uncommon IVD herniation with localization of the disc material, which has not been reported previously. The resected tissue consisted of dura mater and cartilage tissue, suggesting that the disc material had infiltrated the dura mater itself. Based on lesion location, spinal cord lesions are classified as extradural, intradural/extramedullary, or intramedullary (24). According to this classification, the term “intradural/extramedullary” refers to the disc material that breaches the dura and resides in the arachnoid cavity. However, in our case, the disc material did not breach the dura and existed within it, indicating that its localization may represent a true “intradural” herniation.

In cases of acute canine thoracolumbar IVDE, compression and contusion of the spinal cord and nerve roots by disc material can cause pain, paresis or paralysis, and urinary retention or incontinence (25). In our case, the dog exhibited back pain and monoparesis but remained ambulatory, with a neurological grade of 2 at clinical onset. By contrast, dogs with intradural/extramedullary disc herniation are often non-ambulatory (26). Neurological grade is influenced by the degree of spinal compression (27) and has been shown to correlate with white matter damage (28). Clinical signs may be milder in cases involving dural infiltration of disc material than in intradural/extramedullary disc herniation, in which the disc material tears through the dura and directly impacts or compresses the spinal cord.

MRI is the most commonly used modality for diagnosing IVD herniations in dogs (2, 3). Typical MRI findings of IVD herniation include extradural compression of the spinal cord at the level of IVD space, with the herniated nucleus pulposus appearing hypointense within the epidural space on T1WI and T2WI (2). In the present case, MRI revealed a T2WI isointense to hyperintense mass that was intra- or extradural, extramedullary, and showed contrast-enhancement. Spinal meningiomas typically appear as T2WI hyperintense masses contiguous with the meninges and exhibit strong contrast enhancement; however, some cases have been reported as isointense on T2WI as well (29). The MRI findings in this case were initially consistent with those typically observed in meningiomas. These findings closely resembled those of meningiomas. However, it has been reported that extradural compression material and meningeal contrast enhancement can also occur in cases of IVD herniation and should not be interpreted as specific findings of neoplastic lesions (30). Therefore, IVD herniations should be considered as one of the differential diagnoses when such MRI findings are observed. In cases of intradural/extramedullary disc herniation, the Y-sign on MRI has been frequently reported (26). However, in the present case, histopathology confirmed that the disc material did not tear through the dura mater but remained within it. This distinction may explain why the MRI findings differed from those reported for intradural/extramedullary disc herniations.

Initially, meningioma was considered the most likely differential diagnosis, as it was difficult to distinguish between IVD extrusion and meningioma on the first MRI examination alone. However, the second MRI scan provided a critical diagnostic clue—a reduction in the mass size. Meningiomas are not expected to decrease in size spontaneously, whereas IVD material may undergo partial resorption, resulting in a smaller lesion (31). Therefore, conducting a follow-up MRI scan to assess lesion size reduction may be useful in differentiating IVD extrusion with dural infiltration from a meningioma. We also performed CT; however, no abnormal findings were observed. MRI is less likely to result in false-negatives results than CT (2). In this case, the lesion was microscopic, and the higher sensitivity of MRI likely contributed to its detection and the observation of subtle changes. Additionally, differentiating IVD material from meningiomas using cytology alone may be difficult (32); therefore, a definitive diagnosis requires histopathological examination.

The patient had a good postoperative prognosis; therefore, dural infiltration by disc material may have contributed to a good prognosis after surgery. In IVDE, pain is often caused by compression and contusion of the spinal cord and nerve roots (25). In the present case, MRI indicated only mild spinal cord compression, suggesting that the back pain may have resulted more from contusion than from compression. Additionally, pathological examination revealed macrophage infiltration and hemosiderosis around the cartilage tissue, suggesting intradural hemorrhage and inflammation. These findings suggest that inflammation and hemorrhage of the dura mater may also have contributed to the pain and that surgical removal of the thickened dura mater likely played a role in pain relief.

In conclusion, this case represents the first report of a “true intradural” IVD herniation in a dog characterized by the dural infiltration of IVD. When T2WI MRI reveals thickened meninges and contrast-enhanced lesions, both meningiomas and IVD herniation should be considered in the differential diagnoses. Follow-up MRI can aid in distinguishing IVD herniation from meningiomas and other neoplastic diseases. Given the unique localization of the disc material in this type of IVD herniation, surgical resection of the thickened dura mater may contribute to symptom relief and a favorable prognosis.

## Data Availability

The original contributions presented in the study are included in the article/Supplementary material, further inquiries can be directed to the corresponding author/s.
